# The theory behind the strategies: interpreting the expert recommendations for implementing change taxonomy through four behavioural lenses

**DOI:** 10.3389/frhs.2026.1800608

**Published:** 2026-04-02

**Authors:** Per Nilsen, Kristin Thomas, Hanna Augustsson Öfverström, Maria Fagerström, Kathrine Hald, Jeanette Wassar Kirk

**Affiliations:** 1 Department of Health, Medicine and Caring Sciences, Linköping University, Linköping, Sweden; 2 Department of Learning, Informatics, Management and Ethics, Karolinska Institute, Stockholm, Sweden; 3 Danish Center for Health Services Research, Department of Clinical Medicine, Aalborg University, Aalborg, Denmark; 4 Medical Diagnostic Centre, University Research Clinic for Innovative Patient Pathways, Silkeborg Regional Hospital, Silkeborg, Denmark; 5 Department of Clinical Research, Hvidovre University Hospital, Copenhagen, Denmark; 6 Department of Health and Social Context, National Institute of Public Health, University of Southern Denmark, Odense, Denmark

**Keywords:** behaviour change, behaviourism, culture, dual-process models, ERIC taxonomy, implementation strategies, social cognition, theory

## Abstract

**Background:**

Implementation strategies are essential for promoting the uptake of evidence-based practices, yet they are often applied without explicit attention to their theoretical foundations. The Expert Recommendations for Implementing Change (ERIC) taxonomy identifies nine strategy categories, but work on exploring the assumptions underlying how these strategies bring about behaviour change is limited. This study aimed to clarify the theoretical bases of ERIC strategies to strengthen conceptual understanding and guide strategy selection.

**Methods:**

We conducted a conceptual analysis of the nine ERIC strategy categories and examined how each aligns with four major perspectives on behaviour change: behaviourism, social cognitivism, dual-process models, and culture. We identified the implicit assumptions about change processes for each category and interpreted these through the four lenses to compare convergences and divergences in their explanatory mechanisms.

**Results:**

Each theoretical perspective highlighted distinct yet complementary pathways through which implementation strategies operate. Behaviourism emphasized reinforcement and environmental cues; social cognitivism focused on self-efficacy, motivation, and social learning; dual-process models distinguished between automatic and reflective cognitive systems; and cultural perspectives underscored the influence of shared norms and values. Mapping ERIC categories through these perspectives revealed overlaps and tensions, such as between extrinsic reinforcement and intrinsic motivation, or between individual-level processes and collective cultural alignment.

**Conclusions:**

Implementation strategies are not theory-neutral but rest on implicit assumptions about how behaviour changes. Clarifying these assumptions reveals why strategies vary in effectiveness across contexts and provides a foundation for more deliberate, theory-informed strategy selection and evaluation. Integrating behavioural, cognitive, and cultural perspectives offers a multidimensional understanding of change processes, enabling researchers and practitioners to design strategies that are contextually aligned, theoretically coherent, and more likely to produce sustainable outcomes.

## 1 Introduction

Efforts to improve patient and population health rely on implementation strategies to promote the uptake of evidence-based practices in healthcare and beyond. Implementation strategies are “methods or techniques used to enhance the adoption, implementation and sustainability of a clinical program or practice” (1). In essence, they represent the “how” of translating evidence into practice. These strategies, ranging from training to financial incentives and engaging opinion leaders, have been mapped in frameworks such as the Expert Recommendations for Implementing Change (ERIC) (1) and Cochrane's Effective Practice and Organisation of Care (EPOC) (2). However, despite the importance of strategies, they are often selected, applied, and evaluated without explicit attention to the theoretical assumptions on which they are based (3–5). As a result, it is frequently unclear “why” particular strategies should work (6).

The lack of theorizing is relevant because implementation strategies are built on assumptions, whether explicit or implicit, about the factors that influence existing behaviours and drive behaviour change. For instance, training strategies typically assume that increasing knowledge, skills, motivation and self-efficacy will influence implementation behaviours (7). Although previous work has produced valuable taxonomies of strategies, there is limited clarity about the theoretical foundations of the commonly used strategies. Reviews and empirical studies (5, 8, 9) typically describe what strategies are used and with what effect, but less often connect these strategies to underlying theories. While limited theorizing contributes to fragmented knowledge, it is not the sole explanation for heterogeneity in implementation findings. Variability in contexts, measurement approaches, strategy operationalization and reporting practices also play important roles. However, insufficient articulation of theoretical assumptions limits our ability to compare studies meaningfully, synthesize findings across contexts, and refine strategies based on cumulative learning.

This study addresses this gap by examining four theoretical perspectives that underlie each of the nine implementation strategy categories in the widely used ERIC taxonomy (1). By unpacking the assumptions and processes hypothesized behind these strategies, we seek to clarify their theoretical foundations. In doing so, we aim to provide guidance for strategy selection and contribute to a more cumulative understanding of how implementation strategies produce their effects. Our approach complements, rather than replaces, existing strategy selection methods such as Implementation Mapping (10) and the Implementation Research Logic Model (11). Whereas those approaches support structured linking of determinants, strategies, and outcomes, our analysis focuses on clarifying the implicit theories of change embedded within strategies themselves. By making these assumptions explicit, we provide an additional lens that can be integrated into existing planning tools to enhance theoretical coherence.

## 2 Methods

We conducted a conceptual analysis of the nine ERIC implementation strategy categories (1, 12) and examined how each aligns with four major theoretical perspectives on behaviour change: behaviourism, social cognitivism, dual-process models, and culture. A summary of the four theoretical perspectives is presented in Table 1. We identified the core assumptions about how behaviour changes for each category, then interpreted these through each theoretical lens. Strategies emphasizing training or feedback were considered in relation to behaviourist principles, such as reinforcement; those involving peer influence or self-efficacy were examined from a social-cognitive standpoint; strategies using prompts or decision supports were analysed through dual-process models; and those engaging organizational values or norms were considered from a cultural perspective. This approach allowed us to clarify the theoretical underpinnings of each ERIC category and to identify where different perspectives converge or diverge.

**Table 1:** Summary of the four perspectives on behaviour change.

Perspective	Key focus and/or core principles	Processes and characteristics of behaviour change
Behaviourism	Behaviour is learned and maintained through reinforcement; external stimuli and consequences shape actions	Reinforcement and punishment strengthen or weaken behaviours; feedback loops promote repetition of desired actions; cues (stimuli/signals) trigger behavioural responses; learning occurs primarily through conditioning, not cognition
Social cognitivism	Human behaviour is guided by cognitive processes: beliefs, expectations, intentions, and perceptions of control	Observational learning: people learn by watching others; self-efficacy influences persistence and effort; intentions predict behaviour, moderated by subjective norms and perceived control; motivation quality (intrinsic vs. extrinsic) shapes engagement
Dual-process models	Behaviour arises from interaction between automatic (System 1) and deliberate (System 2) processes	System 1: fast, intuitive, habitual responses; System 2: slow, analytical, reflective thinking; behaviour change requires disrupting unhelpful habits and strengthening reflective self-regulation; context and cues can support automatic desired behaviours
Culture	Behaviour is embedded in shared social meanings, norms, and values that define group or organizational life	Culture shapes what behaviours are legitimate or valued; shared symbols and norms guide and constrain action; belonging and identity reinforce conformity to group values; behaviour change often requires cultural alignment rather than individual persuasion

The four perspectives were selected because they frequently frame behaviour change in implementation science (13–17). Together, they provide a comprehensive view of the individual, social, cognitive, environmental and cultural dimensions of behaviour. They address external reinforcements, internal motivations, cognitive processes and collective influences shaped by shared cultural norms (informal rules about acceptable behaviour) and values (core ideals about what is important) within groups, offering a multidimensional framework for understanding and facilitating behaviour change.

Diffusion of Innovations theory (18) shares conceptual ground with these perspectives, particularly in its attention to social influence, network structures, organizational adoption processes and system-level change. Although the diffusion theory is not examined as a separate analytic lens in this paper, it informs several of the perspectives discussed, especially those addressing legitimacy, peer influence and institutionalization, highlighting the interconnected nature of theoretical approaches in implementation science research.

We identified the underlying assumptions of each ERIC strategy category through a systematic, iterative conceptual analysis. The process began with a close reading of the original ERIC definitions and descriptions (1, 12), focusing on implied views of how change occurs, whether through external reinforcement, internal motivation, cognitive reflection, or cultural norms and values.

We then coded key behavioural processes and refined these codes through team discussions to reach a shared interpretation of each strategy's implicit change logic. The assumptions that were identified were subsequently mapped onto the four theoretical perspectives to explore areas of convergence and divergence. This iterative process emphasized interpretation and reflection rather than rigid classification, aiming to reveal the implicit assumptions about behaviour and change that underpin implementation strategies.

### 2.1 Four theoretical perspectives on behaviour change

The understanding of human behaviour has progressed significantly, evolving from early individual-focused theories to more comprehensive perspectives that account for social, cognitive and cultural dimensions. This shift reflects increasing recognition that behaviour is influenced not only by immediate reinforcements or mental processes but also by broader social contexts, including the norms and values of cultural context, whether within a healthcare team, profession, workplace or organization (19).

From a behaviourist perspective, behaviour change is driven by reinforcement principles, where actions are strengthened or weakened based on their consequences (20). Behaviourism was pioneered by John Watson in the early 1990s and later advanced by B.F. Skinner, whose concepts of operant conditioning continue to inform contemporary behavioural analysis (21, 22). Behaviourism highlights the role of corrective feedback loops: reinforcement of desired behaviours increases their likelihood of repetition, whereas the absence of reinforcement or the introduction of negative consequences can diminish unwanted behaviours (23, 24). Cues, defined as stimuli or signals, play a crucial role by prompting specific behaviours and serving as triggers for reinforcement (25, 26).

The cognitive revolution of the 1960s gave rise to social cognitivism, a perspective that emphasized the mental processes neglected by behaviourism. Social cognition focuses on individual cognitions and thought processes that intervene between observable stimuli (e.g., a clinical guideline) and responses (e.g., adhering to the guideline) in real-world situations (e.g., a primary care unit) (27, 28). Key social-cognitive theories include Social-Cognitive Theory (28, 29), which highlights the importance of self-efficacy and observational learning, where individuals learn by observing others rather than solely through direct experience. The Theory of Planned Behaviour (30, 31) focuses on intentions, subjective norms (perceived social pressure to perform or not perform a behaviour), perceived control (belief about how easy or difficult it is to perform the behaviour) and outcome expectations (anticipated consequences of behaviours). Self-Determination Theory (32, 33), another important social-cognitive theory, distinguishes between intrinsic motivation (engaging in activities for their inherent enjoyment or personal satisfaction) and extrinsic motivation (performing tasks to achieve external rewards or avoid punishments) and emphasizes that the quality of motivation is as important as its quantity.

Building on earlier cognitive theories of human behaviour, dual-process models emerged in the 2000s to refine understanding of how thought and behaviour are regulated (34). These models distinguish between two interacting cognitive systems: System 1 is intuitive, fast and automatic, and System 2 is deliberate, slow and analytical (35, 36). System 1 typically governs habitual and routine behaviours, whereas System 2 is activated when individuals encounter complex, unfamiliar or high-stakes situations. For example, routine clinical procedures may be carried out automatically (System 1), whereas diagnosing a new or ambiguous condition requires more deliberate reasoning (System 2) (34, 37). Within this framework, behaviour change involves working with both systems: disrupting unhelpful habits and biases and building supportive contexts and cues that make desired behaviours more automatic, and simultaneously cultivating reflective practices that strengthen awareness, intention and self-regulation (38, 39).

The study of culture has a long history. Early anthropological and ethnographic scholars, including Geertz (40), Spradley (41), and Hammersley and Atkinson (42), pioneered the view of culture as a shared system of meanings that emerges through social interaction. This perspective inspired a surge of interest in organizational culture in the 1980s as a key framework for understanding behaviour within organizations (43, 44). Definitions of culture vary, but most scholars agree it is a learned phenomenon rooted in social contexts, encompassing shared norms and values that define a cultural context, whether a team, workplace, profession, or organization (45, 46). As a fundamentally collective phenomenon, culture can outweigh individual differences, shaping what behaviours are encouraged or discouraged (47, 48). Culture provides a sense of belonging and legitimacy, reinforcing behaviours that align with group norms and values and stigmatizing those that deviate (49, 50).

### 2.2 Theoretical explanations of ERIC implementation strategy categories

The ERIC taxonomy is a highly respected framework within implementation science and was developed to support both research and practice in the field. Published in 2015 after a multi-round expert consensus process, it identifies, defines and categorizes 73 discrete implementation strategies; for example, audit and feedback, educational meetings, facilitation and tailoring to context. These strategies are organized into nine overarching categories, each addressing different aspects of the implementation process. Although the ERIC taxonomy presents strategies as discrete categories, empirical application has shown that strategies often overlap and interact in practice. Our analysis therefore focuses on dominant theoretical logics rather than rigid classification. The widespread adoption and recognition of ERIC underscore its value as a foundational resource for advancing implementation research and practice (1, 12).

The theoretical perspectives of behaviourism, social cognitivism, dual-process models and culture provide lenses for understanding the processes underlying the nine ERIC strategy categories. Each perspective emphasizes distinct pathways through which these strategies influence behaviour change. The analysis considers how each of these theoretical perspectives interprets the nine strategy categories while highlighting areas where their underlying assumptions may conflict. We refer specifically to the ERIC taxonomy unless otherwise stated. Implementation strategies more broadly may extend beyond ERIC.

#### 2.2.1 Use evaluative and iterative strategies

These strategies focus on assessing performance, outcomes or processes and then using that information to refine and strengthen implementation efforts. Examples include audit and feedback, conducting needs assessments (i.e., collecting and analysing information to identify needs, gaps, or discrepancies between current and desired outcomes), and applying Plan-Do-Study-Act cycles (51). The overall purpose is to create a process of continuous learning and improvement by identifying what is working well and what requires adjustment (1).

From a behaviourist perspective, feedback and evaluation function as reinforcements, shaping behaviour by rewarding desired practices and discouraging ineffective ones. In contrast, social cognitivism frames evaluation as a reflective process that supports knowledge-building, self-efficacy and motivation. Dual-process models emphasize how iterative feedback engages slower, reflective reasoning, enabling clinicians to interrupt automatic habits and adopt more deliberate actions. Cultural perspectives highlight how continuous evaluation can institutionalize shared norms and values of learning and accountability, embedding collective reflection and improvement into the culture. Thus, all perspectives view feedback as a catalyst for change, but they emphasize different processes. Behaviourism stresses external reinforcement, social cognitivism and dual-process models highlight internal reflection and cultural perspectives focus on shared norms and values.

#### 2.2.2 Provide interactive assistance

This category involves strategies that offer hands-on, ongoing support to individuals, groups or organizations during implementation. Such support can take the form of facilitation, technical assistance, coaching or mentoring. The aim is to provide practical guidance, encouragement and problem-solving to help overcome challenges and sustain change (1).

In behaviourism, interactive assistance works as immediate reinforcement and correction during practice, strengthening desired behaviours. Social cognitivism interprets assistance as social learning, where individuals gain skills, knowledge and self-efficacy by observing and modelling facilitators, mentors or coaches. From the view of dual-process models, real-time support can interrupt automatic reactions, creating space for more reflective behaviours. Cultural perspectives stress that assistance builds a culture of support where asking for help is normalized and collective problem-solving is valued. Although these perspectives converge on the value of ongoing assistance, they diverge in focus. Behaviourism privileges external reinforcement, social-cognitive theories highlight individual reflection and learning, and cultural views prioritize shared norms and values.

#### 2.2.3 Adapt and tailor to context

Implementation often requires modification to fit the unique characteristics of a particular setting. Strategies in this adaptation and tailoring category emphasize adjusting strategies to local needs, such as customizing workflows, translating or culturally adapting materials or aligning activities with organizational norms. The purpose is to ensure strategies are relevant, feasible and acceptable within different contexts (1).

From a behaviourist standpoint, adaptation and tailoring function by modifying environmental cues to reinforce desired behaviours. Social cognitivism highlights how modification can strengthen self-efficacy and draws on social modelling to encourage change. Dual-process models emphasize that adaptation and tailoring disrupt automatic one-size-fits-all thinking, prompting reflective reasoning about what will work in a specific setting. Cultural perspectives, however, stress that such modifications succeed only when they resonate with the shared norms and values of a team, profession, organization or another group. This points to a potential tension; strategies may empower individuals yet still falter collectively if they conflict with the culture of a group (e.g., a team or profession). In practice, adaptation and tailoring must therefore balance individual-level processes with cultural fit to achieve sustained impact.

#### 2.2.4 Develop stakeholder interrelationships

Strong relationships with key individuals and groups are essential for effective implementation. Strategies in this category include engaging community leaders, building coalitions, fostering consensus, and involving opinion leaders. The purpose is to mobilize collective action, strengthen collaboration, and leverage social influence to enhance adoption, integration, and sustainability of change efforts (1).

From a behaviourist perspective, collaboration facilitates implementation by creating reinforcing conditions that shape and maintain desired behaviours. Recognition, approval, and shared success act as positive reinforcers, encouraging continued participation and alignment with implementation goals. In this view, collaboration functions as an environment of distributed reinforcement that sustains engagement and compliance across actors. Social cognitivism interprets collaboration as a dynamic context for modelling, social learning, and the co-construction of self-efficacy, where stakeholders influence each other's motivation and expectations through interaction. Dual-process models add that trust and repeated interpersonal exchanges provide the psychological safety needed for reflective engagement, allowing stakeholders to move beyond automatic responses and deliberate collectively about complex implementation challenges. From a cultural perspective, collaboration is grounded in shared norms and values of trust, reciprocity, and mutual accountability. Maintaining these cultural foundations ensures that collective action is legitimate and sustainable. Conversely, reducing collaboration to mere reinforcement risks eroding its normative basis and weakening coalitions over time.

#### 2.2.5 Train and educate stakeholders

Knowledge- and skill-building are central to this category. Strategies focus on preparing those involved in or affected by the implementation to carry out their roles effectively. Examples include workshops, academic detailing and the distribution of educational materials. The purpose is to equip stakeholders with the tools and understanding needed to support, adopt and maintain new practices (1).

From a behaviourist perspective, training and education shape behaviour through practice, repetition and corrective feedback, gradually conditioning individuals to develop desired responses. Social cognitivism interprets education as a process of building knowledge, skills, motivation and self-efficacy, often reinforced by observational learning from credible experts. Dual-process models highlight a tension; although training can automatize desired behaviours over time, reflective reasoning must first be engaged to ensure learning is deep rather than superficial. Without this grounding, rote practice risks yielding compliance without durability; for example, handwashing framed merely as supervision-driven rule-following rather than as a means of preventing infection. Cultural perspectives see training as more than skill acquisition, positioning it as a way of embedding shared values and norms of continuous learning within a profession, workplace or organization. Together, these perspectives suggest that training is most effective when it balances repetition with reflection, individual mastery with social modelling and technical skills with cultural integration.

#### 2.2.6 Support clinicians

This category addresses strategies that make clinicians' work easier and more effective because they are often central to implementation. These include providing decision support tools, offering reminders and restructuring roles or workflows to reduce unnecessary burden. The intent is to remove barriers, support clinical practice and encourage adherence to evidence-based approaches (1).

From a behaviourist standpoint, prompts, reminders and decision aids act as cues that trigger desired behaviours. Social cognitivism emphasizes their role in boosting self-efficacy and motivation by reducing uncertainty and building confidence. Dual-process models, however, take a more ambivalent view; although decision aids can reduce reliance on quick, heuristic thinking and encourage more deliberate reasoning, over-reliance on such cues risks deskilling professionals by dampening reflective judgement. In contrast, cultural perspectives frame clinician support less as an individual aid and more as a signal that quality care and staff well-being are priorities in the workplace or organization, thereby reinforcing a culture of support and trust. Together, these perspectives highlight the trade-off between efficiency through routinization and the need to sustain adaptability, professional judgement and cultural alignment.

#### 2.2.7 Engage consumers

Patients, families and the public are increasingly recognized as active partners in supporting implementation. Strategies in this category include patient education, shared decision-making, and public awareness campaigns. The purpose is to empower consumers, strengthen their understanding of interventions and foster active involvement in their own care and in broader system changes (1).

Viewed through a behaviourist lens, involving patients and the public reinforces clinician behaviours through satisfaction and feedback, treating engagement primarily as a source of external reward. Social cognitivism, on the other hand, highlights consumers as active role models and co-producers of health, where observing peers can enhance motivation and shape outcome expectations. Dual-process models suggest that engagement can interrupt reliance on personal heuristics or habits, fostering reflective practices that integrate both clinical evidence and patient values. In contrast, cultural perspectives emphasize that meaningful engagement is grounded in deeply shared norms and values of equity, trust and collective responsibility in decision-making. From this standpoint, reducing engagement to mere reinforcement risks undermining its legitimacy and depth; instead, it is understood as a normative commitment essential to sustaining authentic partnerships.

#### 2.2.8 Utilize financial strategies

Financial considerations can be an important barrier or facilitator of implementation. Strategies in this category include offering monetary incentives, adjusting reimbursement mechanisms, or allocating dedicated funding to support change. The goal is to align financial structures with desired practices, reduce economic barriers and promote long-term sustainability (1).

From a behaviourist standpoint, monetary incentives function as direct reinforcers, increasing the likelihood of desired behaviours. However, Self-Determination Theory from the social cognitivist domain cautions that extrinsic rewards may also undermine intrinsic motivation, eroding autonomy and long-term engagement. Dual-process models further complicate the picture by distinguishing between automatic responses to incentives and more deliberate reflections on their sustainability. Cultural perspectives highlight that financial structures do more than change individual behaviour; they also signal organizational priorities, norms and values, influencing how strategies are perceived and whether they are embraced. What appears behaviourally effective in the short term may, from other perspectives, carry risks of backfiring or misalignment.

#### 2.2.9 Change infrastructure

Some strategies involve altering the broader organizational structures or systems that shape daily practice. This may include revising policies, creating new roles or integrating new practices into existing routines and platforms. The purpose is to build an environment that consistently supports and reinforces the adoption and sustainability of evidence-based practices (1).

Within a behaviourist framework, redesigns can make certain behaviours easier or more rewarding while discouraging others. In social cognitivism, infrastructure is viewed as an enabling environment that fosters opportunities to develop knowledge, acquire skills, adopt behaviours, and establish habits that support evidence-based practice. Dual-process models highlight how structural changes disrupt ingrained habits and routines that are misaligned with evidence-based practice, necessitating a period of reflective adjustment before new, desirable behaviours can become automatic. Cultural perspectives add an additional layer, asserting that behaviours are more likely to take root when they align with the shared norms and values of the relevant group, such as a workplace, profession, or organization. What may appear structurally rational from a behaviourist standpoint can be rejected if it clashes with cultural norms and values, highlighting the potential gap between technical fixes and cultural fit.

## 3 Discussion

### 3.1 Theoretical convergences and tensions in ERIC strategy categories

This theoretical analysis mapped the nine ERIC strategy categories (1) to four major theoretical perspectives, highlighting both convergences and conflicts in how strategies are understood. A central finding is that although all perspectives recognize the value of strategies such as feedback, training, assistance and adaptation, they differ in their assumptions about the processes of change. Behaviourism emphasizes external reinforcement (20), social cognitivism and dual-process models stress internal cognitive processes (52), and cultural perspectives stress the shared norms and values of the group (45, 46). These differences create subtle but important tensions on whether strategies primarily work through individual reinforcement, cognitive reflection or cultural alignment.

Conflicts arise most clearly where theories emphasize divergent loci of change. For example, financial strategies are explained by behaviourism as reinforcers (19, 20) but are critiqued in social cognitivism for potentially undermining intrinsic motivation (32). Similarly, from a behaviourist standpoint, clinician support tools (e.g., checklists) can streamline practice by reinforcing desired behaviours and promoting consistent performance (21). However, viewed through a dual-process lens, over-reliance on such tools may diminish clinicians' engagement in reflective, analytical reasoning, thereby risking the deskilling of professional judgement (34). Cultural perspectives frequently highlight tensions where strategies assumed to work at the individual level may falter if they clash with group norms, values, or legitimacy (45). These conflicts suggest that no one perspective fully captures the processes at play and that combining insights is often necessary.

The analysis of cultural perspectives emphasized shared norms and values, but risks presenting culture as harmonious rather than acknowledging its potential for conflict and inequality (45). Implementation is not only about aligning with norms and values but also about navigating entrenched hierarchies, inequities, and competing professional identities (53). Financial strategies, for example, may reinforce desired behaviours but also exacerbate disparities if incentives privilege well-resourced settings. Similarly, infrastructure changes may align with organizational or professional culture while marginalizing certain groups. Applying the four theoretical lenses can help scholars surface these dynamics by prompting questions about who benefits from reinforcement structures, whose norms are being institutionalized, and which actors are positioned to engage in reflective decision-making. Making such assumptions explicit allows implementation efforts to be designed with greater attention to equity, representation, and power. Future work might consider theories that explicitly address power, identity and equity to avoid reproducing overly technocratic or “neutral” accounts of implementation.

Recent efforts to identify causal mechanisms of implementation (4, 8) share the aim of explaining how strategies produce change. However, these approaches often conceptualize mechanisms in largely context-free terms, such as “knowledge”, “motivation”, or “opportunity”, without explicitly addressing the theoretical assumptions that give these mechanisms meaning. From our perspective, mechanisms cannot be fully separated from the theoretical logics in which they are embedded. What counts as a mechanism, as well as how it is expected to operate, depends on underlying assumptions about human behaviour, cognition, and social context. By making these assumptions explicit through the four theoretical lenses applied here, our analysis complements and extends causal-mechanistic approaches. It emphasizes that identifying mechanisms requires theoretical grounding; mechanisms are not theory-neutral entities but are themselves expressions of particular explanatory traditions.

### 3.2 Applying the four lenses in strategy selection

Applying the four theoretical perspectives to concrete implementation decisions can clarify both the intended mechanisms of action and potential unintended consequences of commonly used strategies. Consider the use of financial incentives to increase guideline adherence. From a behaviourist perspective, incentives function as external reinforcements that strengthen desired behaviours through reward contingencies. A social cognitivist lens, however, raises concerns about potential crowding out of intrinsic motivation if incentives undermine clinicians' sense of autonomy or professional commitment. A dual-process perspective suggests that incentives may encourage routinized compliance without deeper reflective engagement, potentially limiting sustained change once rewards are withdrawn. From a cultural standpoint, incentives signal organizational priorities and may reshape shared norms and values, yet they can also generate perceptions of inequity if rewards are unevenly distributed. Taken together, these lenses suggest that financial incentives may be most effective when combined with reflective educational components and explicit cultural framing that positions adherence as aligned with professional norms and values rather than merely externally rewarded behaviour.

A similar multidimensional analysis can be applied to audit and feedback interventions. Behaviourally, feedback reinforces performance by making discrepancies between current and desired practice visible. Social cognitive theory highlights its role in strengthening self-efficacy when feedback is constructive and attainable goals are provided. From a dual-process perspective, feedback can activate reflective (System 2) processing by prompting deliberate evaluation of practice patterns. At the cultural level, regular audit and feedback may normalize transparency and accountability within teams. However, in highly hierarchical settings, public feedback risks triggering defensiveness or shame. Integrating facilitated group reflection or peer discussion can mitigate these risks and enhance learning-oriented norms.

Checklist implementation offers a further illustration. Behaviourally, checklists cue and reinforce correct procedural steps. Yet from a dual-process perspective, over-reliance on checklists may shift practice toward automaticity, potentially diminishing critical reflection in complex cases. Culturally, checklists may be resisted if framed as bureaucratic oversight rather than professional support. Their successful adoption therefore depends not only on technical design but also on how they are introduced and legitimized within professional communities. Presenting checklists as tools that enhance, rather than constrain, clinical expertise can align behavioural reinforcement with reflective practice and shared professional values.

Across these examples, the four lenses reveal that implementation strategies are rarely theoretically neutral. Making their underlying assumptions explicit allows researchers and practitioners to anticipate tensions, design complementary supports, and adapt strategies to local contexts in a more deliberate and theoretically informed manner.

### 3.3 Strengths and limitations

Like any conceptual analysis, this paper has several limitations that should be acknowledged. It is primarily conceptual and interpretive, which brings both strengths and weaknesses. By drawing on four established theoretical perspectives, we sought to clarify the assumptions underlying ERIC strategies (1). However, the analysis inevitably reflects selective emphasis; other theoretical traditions, such as systems thinking (54) or theories of diffusion and innovation (18), were not systematically included, even though they may shed light on aspects of strategy use that behaviourism, social cognitivism, dual-process models and cultural perspectives do not. In this sense, the analysis is not exhaustive, but rather a step towards building a more integrated theoretical foundation.

A further limitation is that the analysis remains abstract rather than empirical. The paper did not test these theoretical alignments with data from implementation studies. Future empirical research could examine whether and under what conditions particular theoretical perspectives actually predict or explain the effectiveness of specific strategies. Similarly, the analysis focused on categories as defined by the ERIC taxonomy, but in practice, strategies often overlap, interact or evolve in ways that blur categorical boundaries. These limitations underscore that although theory-based analysis provides conceptual clarity, its practical value depends on ongoing testing, refinement and integration with empirical evidence (9).

A strength of this study lies in its comparative framing. By examining each ERIC strategy category (1) across multiple theories and perspectives, the analysis avoids privileging a single explanatory lens from the outset. This side-by-side comparison highlights complementarities and tensions between theoretical perspectives, helping to show where different perspectives converge, where they diverge and how they can be integrated. Such an approach provides decision-makers with more flexibility; rather than applying strategies in a one-size-fits-all manner, they can select and adapt strategies with a clearer understanding of the assumptions and processes that are most relevant in their context.

The study contributes to implementation science by systematically linking the nine ERIC strategy categories (1) to four major theoretical perspectives on human behaviour. Rather than viewing strategies as generic or atheoretical tools, it sheds light on the assumptions that underpin how and why particular approaches produce change. This theory-informed analysis advances the field by clarifying conceptual foundations, guiding strategy selection and evaluation, and fostering cumulative knowledge-building across studies and contexts.

### 3.4 Implications for research and practice

The findings highlight several important implications, particularly regarding how theoretical perspectives are combined in implementation science research. When integrating theories, researchers should aim to leverage complementary insights while remaining mindful of potential conflicts between theoretical assumptions. It is helpful to first identify the primary locus of change emphasized by each theory, such as individual behaviour, cognitive processes, norms, values, or structural factors, and then map how these loci interact within the research context. For example, a behaviourist strategy such as financial incentives could be combined with social cognitivist insights to mitigate risks to intrinsic motivation, or dual-process perspectives could refine strategies designed to streamline practice by safeguarding reflective judgement. Researchers should avoid superficial combinations of theories, which risk creating contradictions or overlooking key limitations. Instead, thoughtful synthesis should address tensions, such as conflicts between individual-level strategies and group norms and values, and explicitly manage these challenges to build a deeper understanding of behaviour change processes.

Our analysis also has implications for perspectives that conceptualize behaviour change techniques (BCTs), such as those articulated in the Behaviour Change Wheel framework (55). BCTs are often described as the smallest active components of interventions, and some scholars view them as overlapping with or functioning as implementation strategies. Although we did not analyse BCTs separately, they can be interpreted through the same four theoretical lenses applied here. For example, techniques such as prompts and incentives align closely with behaviourist principles, whereas techniques targeting beliefs, intentions, or social influence resonate with social cognitivist theories. Other techniques may engage reflective reasoning (dual-process perspectives) or reinforce shared norms and values of a professional group (cultural perspectives). In this sense, the present framework can be applied not only to ERIC strategy categories but also to the micro-level components that comprise complex implementation efforts.

Taken together, these insights have direct implications for strategy selection and design in practice. First, strategy selection should not be approached as a theory-neutral process; instead, practitioners should carefully consider which theoretical assumptions align most closely with their specific context and objectives. This approach is particularly crucial in situations where empirical evidence supporting the effectiveness of strategies is limited or inconclusive. Second, tensions between perspectives can be leveraged productively. For instance, combining reinforcement with reflective training and cultural alignment may produce more durable outcomes than any single approach. Third, implementation science should integrate theoretical pluralism more explicitly, acknowledging that strategies work through multiple processes simultaneously. Importantly, this approach does not require wholesale retraining but can be incorporated into existing planning workflows by making implicit assumptions explicit.

Finally, these findings point to several priorities for future research. Empirical studies should test the theoretical mechanisms proposed here, using mixed-method and comparative designs to identify when reinforcement, reflection or cultural fit matters most. Studies could also explore how strategies interact across levels; for example, how cultural alignment moderates the effectiveness of reinforcement or cognitive-based strategies. More broadly, there is a need to develop integrative models that move beyond juxtaposing perspectives and instead explain how they can be combined to guide strategy design, sequencing and adaptation.

## 4 Conclusion

In conclusion, this theoretical analysis demonstrates that the ERIC strategy categories can be meaningfully interpreted through four theoretical perspectives, each offering distinct insights into processes of behaviour change. Areas of convergence highlight shared recognition of strategies' importance, and conflicts reveal underlying assumptions that shape how strategies are expected to work. These insights underscore the importance of explicitly theorizing implementation efforts, acknowledging tensions and designing strategies that integrate behavioural, cognitive and cultural perspectives. By making theoretical foundations more transparent, the field can strengthen both the science and practice of implementation.

## Data Availability

The original contributions presented in the study are included in the article/Supplementary Material, further inquiries can be directed to the corresponding author/s.
